# Assessment of Chitosan-Based Hydrogel and Photodynamic Inactivation against *Propionibacterium acnes*

**DOI:** 10.3390/molecules23020473

**Published:** 2018-02-22

**Authors:** Maria Lucia Frade, Sarah Raquel de Annunzio, Giovana Maria Fioramonti Calixto, Francesca Damiani Victorelli, Marlus Chorilli, Carla Raquel Fontana

**Affiliations:** 1 Department of Clinical Analysis, School of Pharmaceutical Sciences, São Paulo State University (UNESP), 14800-903 Araraquara, São Paulo, Brazil; marifrad@hotmail.com (M.L.F.); sarinha_annunzio@hotmail.com (S.R.d.A.); 2 Department of Drugs and Medicines, School of Pharmaceutical Sciences, São Paulo State University (UNESP), 14800-903 Araraquara, São Paulo, Brazil; giovana.calixto@gmail.com (G.M.F.C.); francescavictorelli@gmail.com (F.D.V.); chorilli@fcfar.unesp.br (M.C.)

**Keywords:** chitosan hydrogel, photodynamic therapy, methylene blue, *Propionibacterium acnes*

## Abstract

Chitosan (CH) is a biopolymer that exhibits a number of interesting properties such as anti-inflammatory and antibacterial activity and is also a promising platform for the incorporation of photosensitizing agents. This study aimed to evaluate the efficacy of antimicrobial activity of chitosan hydrogel formulation alone and in combination with the methylene blue (MB) associated with antimicrobial photodynamic therapy (aPDT) against planktonic and biofilm phase of *Propionibacterium acnes*. Suspensions were sensitized with 12.5, 25.0, 37.5, 50.0 μg/mL of MB for 10 min and biofilms to 75, 100 and 150 μg/mL for 30 min then exposed to red light (660 nm) at 90 J/cm^2^ and 150 J/cm^2^ respectively. After treatments, survival fractions were calculated by counting the number of colony-forming units. The lethal effect of aPDT associated with CH hydrogel in planktonic phase was achieved with 12.5 µg/mL MB and 1.9 log_10_ biofilm reduction using 75 µg/mL MB. Rheological studies showed that formulations exhibited pseudoplastic non-Newtonian behavior without thixotropy. Bioadhesion test evidenced that the formulations are highly adhesive to skin and the incorporation of MB did not influence the bioadhesive force of the formulations.

## 1. Introduction

Acne vulgaris exemplifies a disease whose main bacterium related to its etiology is the Gram-positive *Propionibacterium acnes* (*P. acnes*). This bacterium is commonly isolated from areas of skin rich in sebum and when there is an increase of its production in the pilosebaceous unit, there is a greater proliferation of the bacterium which thus behaves as an opportunistic microorganism. When the infectious process is installed and follicle rupture occurs, *P. acnes* can reach more superficial layers of the skin such as the epidermis, characterizing the disease [1].

This dermatosis is mainly observed in adolescents and young adults, being more prevalent in males and more precocious in female adolescents [2]. The disease can persist in 50% of cases in adulthood, and can leave deep scars. Although the disease is not associated with severe morbidity, it causes patients significant psychological repercussions, among them, low self-esteem, social inhibition, depression and anxiety, mainly due to scarring that can be minimized with appropriate early treatment [3]. Currently, there are a large number of acne treatment options available to patients. The disease has been treated in recent decades with antimicrobials such as erythromycins, clindamycins and tetracyclines and with topical and systemic retinoids. However, adverse effects of the use of retinoids and resistance to systemic antimicrobials reaching high levels make their use restricted, emphasizing the need for alternative treatment options [4].

Antimicrobial Photodynamic Therapy (aPDT) is a new treatment modality that has been extensively studied. Its mechanism of action consists of the activation of a drug called photosensitizer by a light source. The photosensitizer is a molecule capable of absorbing light energy at a specific wavelength and transferring it to the surrounding oxygen molecules or biomolecules, generating singlet oxygen or free radicals, causing damage to the microorganism or to target cells [5].

One of the photosensitizers that is already heavily employed in studies involving aPDT in vitro [6,7] and in vivo [8] is methylene blue (MB). Its use has many advantages such as a high quantum yield, high water solubility, absorption of light at red wavelengths that allows great penetration of light in the tissues, becoming one of the most used photosensitizers in both in vitro and in vitro PDT studies [9].

Drug delivery systems have represented promising platforms for incorporation of photosensitizing agents employed in aPDT [10]. Among them, hydrogels occupy a prominent position. These are a three-dimensional polymer networks capable of absorbing large amounts of water while remaining insoluble due to interconnections between the individual polymer chains [11]. 

Chitosan is a natural, high molecular weight polycationic linear polysaccharide. It is derived from chitin, the main structural component of arthropod exoskeletons (including crustaceans and insects), diatoms and seaweed, and also contributes to the mechanical strength of some fungal cell walls [12]. Due to its bioadhesion and permeability properties, it is able to form a hydrogel that can be used as a drug release systems for topical and oral use, being a good candidate as an antibiotic and photosensitizer releasing agent [13]. In addition to being an interesting option as a local drug delivery system, the antimicrobial action of chitosan itself may increase the bactericidal effect of therapy [14]. 

Studies have shown that chitosan has anti-inflammatory and antioxidant activity, antimicrobial effects, and is also used as a drug delivery system, among other important functions in the biological field [15]. Its antimicrobial activity has been explained by several theories [16]. The most accepted hypothesis is that its action on bacteria would occur by the loss of its intercellular components. Due to its positive charge, it binds to the bacterial membrane causing a change in permeability and the output of its intercellular components, thus leading to cell death [17]. The antimicrobial activity of chitosan was observed against a wide variety of microorganisms, including fungi, algae and bacteria, being more active against Gram-positive bacteria than Gram-negative ones [18].

The large water content and broad pores of most hydrogels often generates a relatively rapid release of the drug [19]. This is an important feature in the choice of such formulation, considering that the concentrations of the photosensitizers and the light doses applied are dependent on these fundamental properties for the success of photodynamic therapy.

In this context, the present study aimed to evaluate the in vitro efficacy of a chitosan hydrogel formulation alone and chitosan hydrogel doped with methylene blue as a photosensitizer to be associated with aPDT under irradiation of a red light emitting system (660 nm) against *P. acnes* in planktonic and biofilm phase.

## 2. Results

### 2.1. Structural Characterization of Formulations

**Figure 1:**
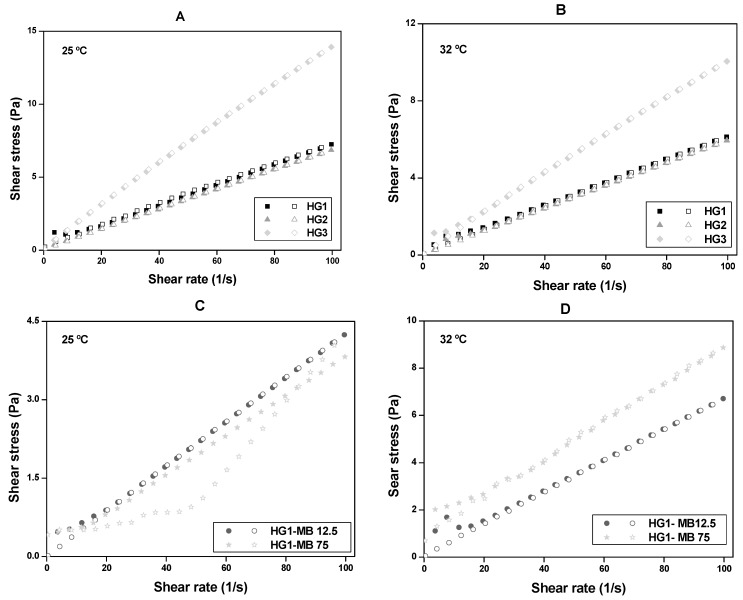
Flow rheograms of: (**A**) HG1, HG2 and HG3 at room temperature (25.0 ± 0.5 °C); (**B**) HG1, HG2 and HG3 at skin temperature (32.0 ± 0.5 °C); (**C**) HG1-MB12.5 and HG1-MB75 at room temperature (25.0 ± 0.5 °C) and (**D**) HG1-MB12.5 and HG1-MB75 at skin temperature (32.0 ± 0.5 °C). Notes: The flow properties were performed using a controlled shear rate procedure ranging from 0.01 to 100 s^−1^ (or ascendent curve—filled symbols) and back (or descendant curve—empty symbols). Standard deviations have been omitted for clarity; however, in all cases, the coefficient of variation of triplicate analyses was less than 10%.

Flow behaviors of CS at room temperature (25.0 ± 0.5 °C) and skin temperature (32.0 ± 0.5 °C) are illustrated in the rheograms in Figure 1. Table 1 shows the the consistency (*k*) and flow (*η*) indexes that were determined from the power law described in Equation (1) for a quantitative analysis of flow behavior:

τ = *k*·γ*^η^*, (1)

where τ is the shear stress, γ is the shear rate, *k* is the consistency index, and *η* is the flow index.

The results showed that all formulations, independent of temperature, exhibited pseudoplastic Non-Newtonian behavior without thixotropy because they presented *η*-values less than unity (*η* < 1) and the upward curves overlapped the downward curves. It was also observed that the *k* of HG3 was remarkably higher than the HG1 and HG2 (Table 1) at both temperatures, showing that the viscosity of the formulation is affected by CS concentration. 

**Figure 2:**
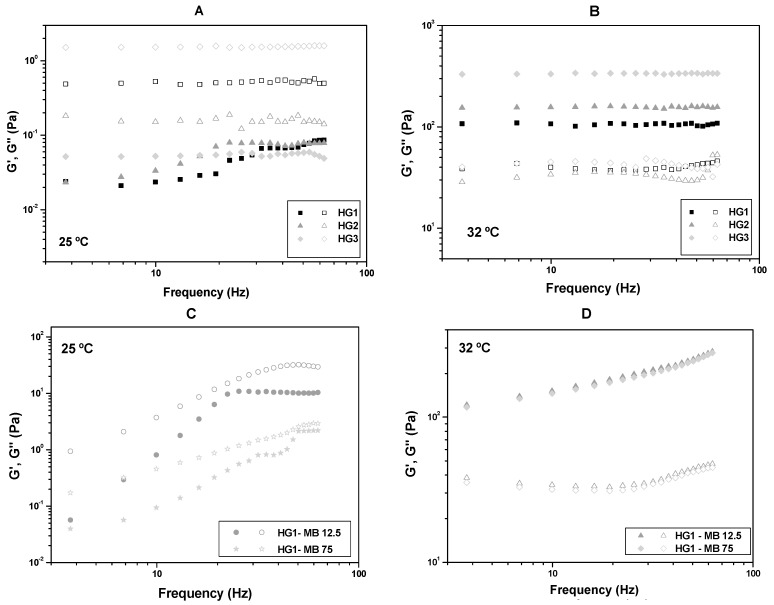
Frequency sweep profile of (**A**) HG1, HG2 and HG3 at room temperature (25.0 ± 0.5 °C); (**B**) HG1, HG2 and HG3 at skin temperature (32.0 ± 0.5 °C); (**C**) HG1-MB12.5 and HG1-MB75 at room temperature (25.0 ± 0.5 °C) and (**D**) HG1-MB12.5 and HG1-MB75 at skin temperature (32.0 ± 0.5 °C). Notes: The storage modulus G’ are the filled symbols and the loss modulus G” are the empty symbols.The SDs have been omitted for clarity; however, in all cases, the coefficients of variation of the triplicate analyses were less than 10%.

The oscillatory analyses of HG1, HG2 and HG3 at room temperature (25.0 ± 0.5 °C) and skin temperature (32.0 ± 0.5 °C) are shown in Figure 2A,B, in which the storage modulus G’ and the loss modulus G” are plotted against the frequency. All data indicated that the elasticity of the formulations increases with increasing temperature. 

Table 2 shows the formulation strength (*S*) and the viscoelastic exponent (*n*) obtained using Equation (2):

G’ = *S*.ω*^n^*, (2)

where ω is the oscillation frequency.

Table 1, as well as Figure 1C,D, show that HG1-MB12.5 and HG1-MB75 continued to present pseudoplastic Non-Newtonian behavior (*η* < 1) at both temperatures. However, the incorporation of MB decreased the consistency of both HG1-MB12.5 and HG1-MB75 at 25 °C, indicating that the MB may have interfered in the formulation network. On the contrary, at 32 °C, MB increased the consistency of HG1. 

Furthermore, the oscillatory data from Figure 2C,D show that MB did not alter the viscoelasticity property of HG1-MB12.5 and HG1-MB75 because both formulations became elastic at 32 °C, like HG1.

**Figure 3:**
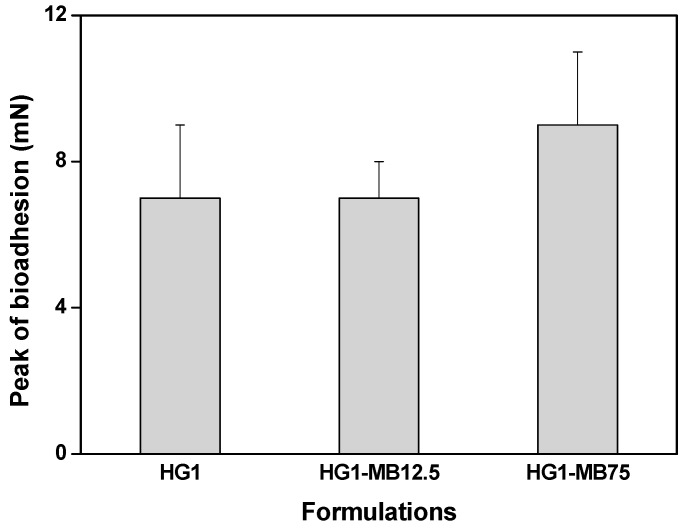
The peak of bioadhesion of the formulations HG1, HG1-MB12.5 and HG1-MB75. Each value represents the mean ± standard deviation of four replicates. The data were collected at 32 ± 0.5 °C.

The peak bioadhesive force between the formulations (HG1, HG1-MB12.5 and HG1-MB75) and the skin is illustrated in Figure 3. The incorporation of MB did not influence the bioadhesive force (*p* > 0.001) of the HG1.

### 2.2. Photodynamic Therapy Studies

**Figure 4:**
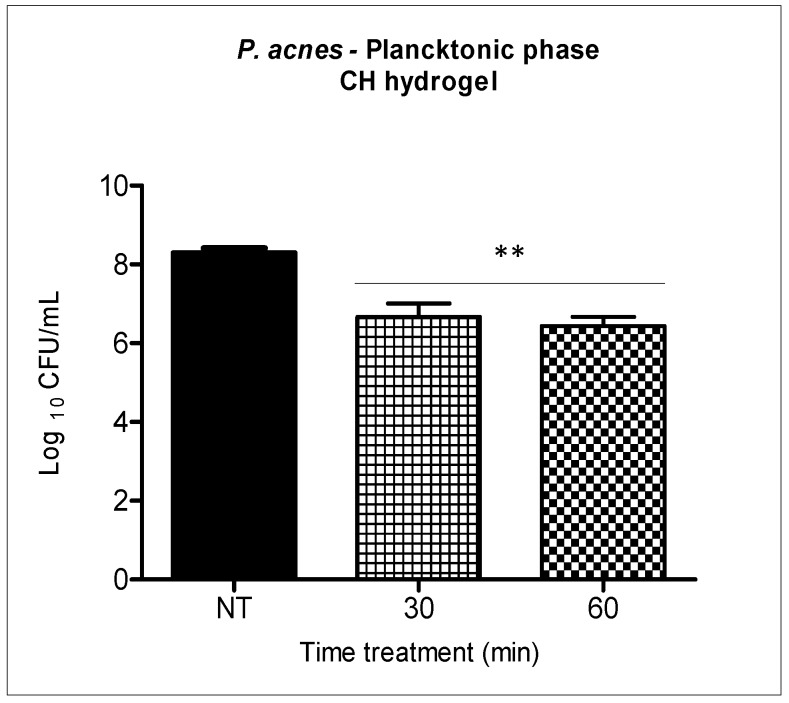
Bacterial suspension incubated with the chitosan hydrogel at 0.25% for thirty and sixty minutes. Columns represent the average of three independent assays and bars represent the standard deviation. The asterisks indicatewhere there is a significant difference in comparison with the groups treated and the group no treated (NT) (one-way ANOVA with Tukey’s post-hoc). ** *p* < 0.01.

**Figure 5:**
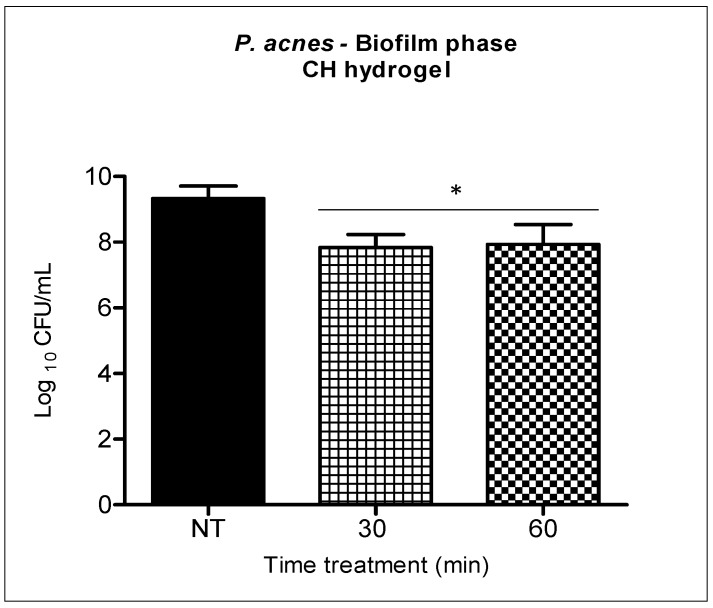
*P. acnes* biofilm incubated with the chitosan hydrogel at 0.25% for thirty and sixty minutes. Columns represent the average of three independent assays and bars represent the standard deviation. The asterisks indicate the statistical difference between the groups and the group no treated (NT). (One-way ANOVA with Tukey’s post-hoc). * *p* < 0.05.

The antimicrobial activity of the chitosan hydrogel against *P. acnes* was evaluated separately at two different incubation times (30 and 60 min) for both suspensions (Figure 4) and biofilms of *P. acnes* (Figure 5). 

Chitosan evaluated separately reached a reduction of 1.9 log_10_ when the bacterial suspension was incubated with the chitosan hydrogel at 0.25% for thirty minutes, and a reduction of 2 logs_10_ was achieved in sixty minutes. These results indicate that there was no statistical difference between the two different analyzed incubation times.

In the biofilm, the reductions were 1.5 log_10_ and 1.4 log_10_ after thirty and sixty minutes of chitosan hydrogel incubation respectively. The two times tested with CH provided statistically significant bacterial reduction (*p* < 0.05) in relation to the untreated group, however, there was no statistical difference between the two treatment times (Figure 5).

**Figure 6:**
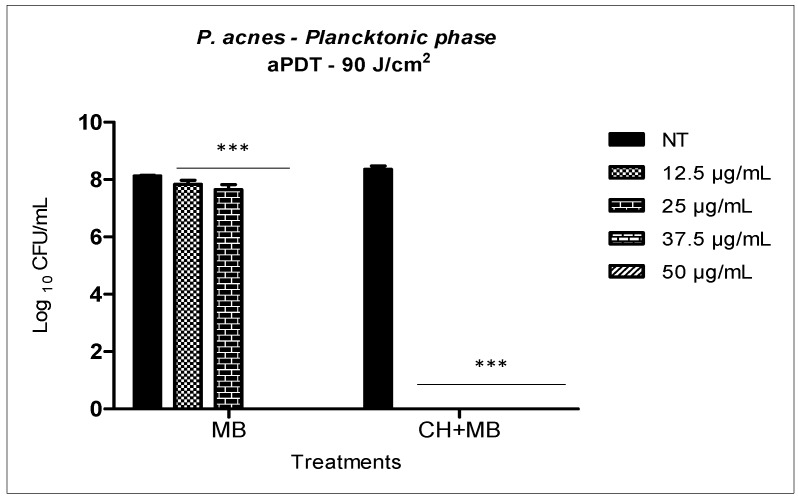
aPDT mediated by MB in solution and MB concentrations incorporated into 0.25 chitosan hydrogel over the standard suspension of *P. acnes*. Columns represent the average of three independent assays and bars represent the standard deviation. The asterisks indicate where there is a statistical difference in comparison with the groups treated and the group no treated (NT); (two-way ANOVA with post-test Bonferroni), *** *p* < 0.001.

Methylene blue solution associated with red LED system at 90 J/cm^2^ fluence against *P. acnes* in planktonic phase, showed a reduction of 0.3 log_10_ when the concentration was 12.5 μg/mL of MB and 0.4 log_10_ with 25 μg/mL of MB. However, increasing MB concentration to 37.5 μg/mL and 50 μg/mL, it was possible to obtain the total reduction of the microbial load, reaching a reduction of 8.1 log_10_ (Figure 6). When the same MB concentrations were incorporated into 0.25% chitosan hydrogel (CH), still in the planktonic phase; it was possible to note the synergistic effect involving the antimicrobial action of chitosan and aPDT. In the CH + MB mediated aPDT, a three-fold lower concentration of PS was required when compared to aPDT with MB in solution. The synergistic effect of aPDT with chitosan hydrogel provided 8.3 logs_10_ reductions with only 12.5 μg/mL of MB.

**Figure 7:**
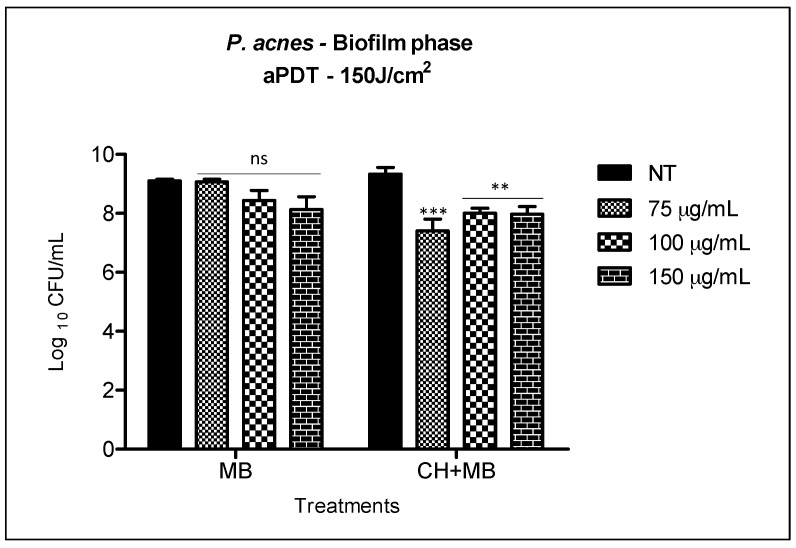
aPDT mediated by MB in solution and MB concentrations incorporated into 0.25 chitosan hydrogel over biofilm of *P. acnes*. The columns represent the average of three independent assays and bars represent the standard deviation. The asterisks represent the statistical difference between the groups treated and the group no treated (NT)(two-way ANOVA with post-test Bonferroni).** *p* < 0.01; *** *p* < 0.001.

The bacterial reduction observed with MB solution associated with irradiation at 150 J/cm^2^ against *P. acnes* biofilm, was not statistically significant for the three concentrations evaluated (75, 100 and 150 μg/mL). The highest biofilm reduction was found when 150 μg/mL of MB (0.9 log_10_) was applied. Once the therapy was associated to chitosan hydrogel doped with methylene blue, all the three concentrations tested increased biofilm reduction. We observed statistically significant reduction when comparing the two treatments (1) MB with (2) CH + MB (Figure 7).

However, the reduction observed in (2) CH + MB (1.9 log_10_ with 75 μg/mL and 1.3 log_10_ with both 100 and 150 μg/mL of MB) was very similar to that obtained when we treated biofilm only with chitosan hydrogel (Figure 5). Thus, we consider there is no synergism with the two antimicrobial therapies in the treatment of *P. acnes* biofilm.

**Figure 8:**
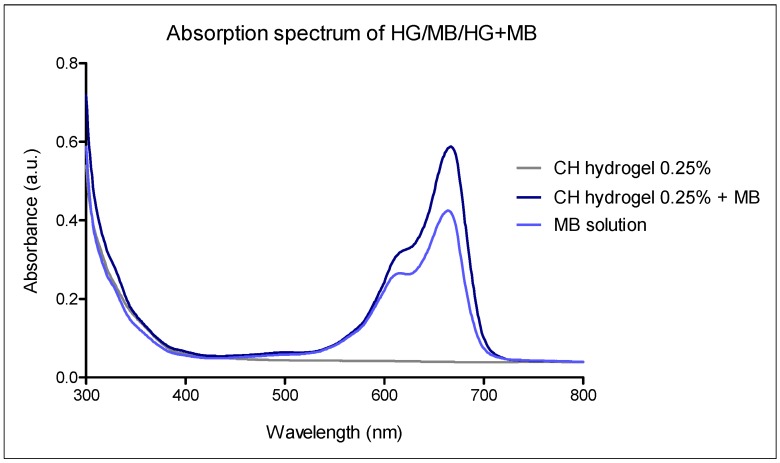
Absorption spectrum of 0.25% chitosan hydrogel with methylene blue.

The absorption spectrum of 0.25% chitosan hydrogel with methylene blue was performed to make sure that the hydrogel would not cause the methylene blue absorption band shifts. As can be seen in Figure 8, the absorption spectrum of MB was unaffected when it was incorporated into the hydrogel, thus not compromising the effectiveness of the photodynamic therapy.

## 3. Discussion

The skin at times is afflicted with a variety of inflammatory and non-inflammatory disorders. One such disorder is acne, a multifactorial common disease that comprises lesions of various skin morphologies, ranging from non-inflammatory (comedones) to inflammatory lesions such as a pustule, papule, nodule, or cyst depending on the depth in the dermis and degree of inflammation.

Briefly, acne vulgaris develops as the result of an interplay of the following factors: (1) follicular epidermal hyper proliferation with subsequent plugging of the follicle; (2) excess sebum production; (3) the presence and activity of the commensal *Propionibacterium acnes* bacteria; and (4) inflammation [20].

Considering the major goals of acne treatment are the resolution of inflammatory lesions, prevention of persistent inflammation and comedones formation and assuming the growing occurrence of antibiotic-resistant bacteria and the severity of the consequences of this trend it is appropriate to maximize use of non-conventional antimicrobial therapy when treating acne [21].

Chitosan and aPDT are non-conventional alternative antimicrobial therapies and therefore, the purpose of this work was to evaluate the effectiveness of photodynamic therapy against *P. acnes* infection using a biomaterial that has, in addition to its own antimicrobial properties, the ability to serve as a vehicle for the administration of the photosensitizer for the accomplishment of combined therapies.

Chitosan may be a good alternative in this treatment because it exhibited anti-inflammatory properties, intrinsic antibacterial activity, analgesic effect and prevention of scar formation [22]. As a non-toxic, biocompatible and biodegradable biopolymer [23], chitosan has not been shown to cause irritation or foreign body reactions at the application sites and is therefore considered a safe product. Chitosan has demonstrated antimicrobial activity against different pathogens, including *Staphylococcus aureus*, *Staphylococcus epidermidis*, *Pseudomonas aeruginosa*, *Escherichia coli*, *Streptococcus mutans*, *Helicobacter pylori* and *Porphyromonas gingivalis* [13,14,18,24]. The mechanism of chitosan’s action is not fully elucidated, but it is known that the interaction of the polymer with the microorganisms occurs at the cell surface level, compromising the wall or cell membrane integrity. Therefore, it can be stated that the degree of microbicidal activity of chitosan is closely related to the characteristics of the cell surface. In Gram-positive bacteria, lipoteichoic acids are the probable responsible for the connection of the chitosan, leading to the destabilization of the cellular membrane and consequently to the extravasation of the intracellular content [16]. Chitosan-based hydrogels are well studied for applications in biomedical fields, especially in wound treatment, tissue engineering and as a drug delivery system [25].

Considering that any factors such as chitosan (CS) concentration, MB incorporation and temperature, can modify the physicochemical properties of the hydrogels, rheological and bioadhesive studies were performed to characterize the formulations.

Our findings showed that the formulations have pseudoplastic and non-thixotropic characteristics that are suitable for skin drug delivery systems because both behaviors facilitate the formulation application. It occurs due to the hydrogel network arranges itself in the path of shear, reducing the formulation viscosity with increasing the shear rate. Hence, the formulation begins easily flowing on the skin surface, but the removal of shear stress results in the fast recovery the internal structure of the hydrogel and returning the viscosity to the original state, helping the formulation fixation on the skin, because it will not leak [26].

Moreover, as the chitosan concentration increased, the viscosity of the formulation increased. The CS dispersion presents in the form of quasi-globular conformation stabilized by extensive intra- and inter-molecular hydrogen bonding; then the increase of amine and hydroxyl groups can affect the rigidity of the polymer film and cause the high viscosity of chitosan dispersion [27].

The oscillatory analyses of HG1, HG2 and HG3 showed that these formulations are more viscous than elastic (G” > G’) at 25 °C indicating a weak network consisting of physically entangled systems, while with increasing temperature to 32 °C, they become elastics, showing a strong network consisting of secondary bonds [28].

Furthermore, the increase of the formulation strength can be attributed to the presence of PO polymer that is a polyoxyethylene copolymer that undergoes gelling when the temperature is increased. This transition from the sol phase to gel occurs due to the dehydration of the hydrophobic block of PO resulting in polymer aggregates into micelles with high viscosity [29].

CS increasing also altered the viscoelastic properties of the formulations. It can be suggested that the increase in the CS concentration forms elastic formulations with strong H-bonds between the amines groups improving the interactions between the polymer networks [28].

There is no standard formulation available for topical drug delivery systems. However, it is well known that very elastic formulations can influence the interaction between the formulation and the biological substrate because there are few sites of interaction between the polymers and the skin proteins. In addition, high polymer concentrations may also greatly delay the release of the drug, which does not meet our goal [30].

Therefore, HG1 showed to be the most suitable formulation to be applied on the skin surface since it presented the useful flow behavior, besides to be slightly elasticity at 32 °C; hence, HG1 was selected to proceed with rheological studies in order to observe if the MB can influence its rheology characteristics [31]. For this, both concentrations of MB (12.5 µg/mL and 75.0 µg/mL) was incorporated into HG1, being the formulations named HG1-MB12.5 and HG1-MB75, respectively. 

The set of results from continuous and rheological studies indicated that HG1-MB12.5 and HG1-MB75 maintain the great rheological properties of HG1 at 32 °C, helping in the clinical performance of the treatment. 

The main advantage of the bioadhesive drug delivery systems-based is to prolong the residence time of the drug at the application site, which allows for enhanced contact of the formulation with the biological barrier, decreasing the frequency of application of the product and, thereby increasing patient adherence to the treatment [32].

The bioadhesion test demonstrates that the MB incorporation did not influence the interaction adhesive between the HG1 and the skin. In addition, the found bioadhesion values are close to the values found by Cintra et al. [33], who also developed skin delivery systems.

Our planktonic results showed significant microbial reduction caused by chitosan hydrogel when incubated for 30 and 60 min. Similarly, Friedman et al. [34] also demonstrated antimicrobial activity of chitosan against *P. acnes* in the planktonic phase. However, we observed that there was no complete elimination of *P. acnes* after 30 and 60 min of incubation (Figure 4).

In addition to the surface characteristics of the microorganisms, there are several factors that may interfere with the antimicrobial activity of chitosan inherent in the polymer itself, such as molecular weight, positive charge density, hydrogel concentration, and external factors such as ionic strength and pH, being able to justify the results found in planktonic phase [28]. 

In this study, trying to improve the biofilm reduction, hydrogels containing 0.5 (HG2) and 1% (HG3) of chitosan were also initially tested. However, they were discarded from the continuity, because the higher their concentration in the hydrogel, the more viscous it becomes, making it difficult to perform the biofilm tests in the adopted methodology, as can be seen on rheology assays. 

Since chitosan concentration of 0.25% in planktonic phase and biofilm revealed reasonable bacterial reduction, it was used in the study to evaluate MB-mediated synergism with aPDT. In addition, the use of the standardized culture medium (TSB) caused the chitosan to precipitate in the hydrogel, requiring the modification of the culture medium for the chitosan hydrogel assays.

We carried out experiments with culture medium whose chemical composition was as simple as possible and would cause less interference with the hydrogel. Thus, our results demonstrated that the use of the Mueller-Hinton broth remedied the precipitation problem, possibly by modifying the ionic strength of the medium.

The final pH of the chitosan solution may also decrease its antimicrobial activity, mainly due to the fact that its molecule becomes polycationic at pH lower than its p*K*_a_ (6.3–6.5) [35]. It has been reported that chitosan at pH greater than 7.0 loses the positive charge of the amino groups of the molecule, impairing its antimicrobial capacity. This may have contributed to the results of the present work since the pH of the culture medium used in this study is close to 7.3.

In order to optimize the antimicrobial effect, the 0.25% chitosan hydrogel incorporated with different concentrations of MB was used for a possible synergistic effect after photodynamic therapy.

Considering the results obtained in *P. acnes* planktonic phase (Figure 6), it was possible to observe that the photodynamic therapy mediated by methylene blue (from 12.5 to 50 μg/mL) caused significant statistical reductions and a complete elimination of *P. acnes* was obtained with MB at concentrations of 37.5 μg/mL. When MB was incorporated into the chitosan hydrogel, we observed a synergistic effect (aPDT + CH), since all concentrations of MB incorporated into the hydrogel caused complete elimination of *P. acnes*. The synergistic effect of aPDT + chitosan allowed the use of the photosensitizer (MB) at a concentration three times lower than the concentration used in the monotherapy.

These results in the planktonic phase suggest that the action of chitosan on the *P. acnes* membrane may have facilitated the internalization of PS in the bacterium, requiring lower concentrations of the same for the elimination of the microbial load during photodynamic therapy.

Methylene blue, when excited by a light at a suitable wavelength, reacts with nitrogenous bases of the DNA, specifically guanine, to form 8-hydroxyl-2-deoxyguanosine. This product may lead to the formation of adducts and breaks in the cellular DNA chain [24].

In the biofilm, the results showed a great difference in comparison to those obtained in the planktonic phase. The ability to form biofilms of *P. acnes* is well established in the literature and has been demonstrated in in vitro studies [36,37] and in vivo [38,39]. This mode of cellular organization can be considered the key factor in the pathogenesis of acne since the biofilm formed by *P. acnes* contributes to the formation of an adhesive glue that leads to the attachment of corneocytes resulting in micro-cysts [40].

Coenye et al. [41] demonstrated that *P. acnes* cells involved in biofilms were more resistant to antimicrobial agents compared to planktonic cells, producing a greater amount of extracellular lipases. This fact may explain the number of failed antibiotic therapies in the treatment of acne vulgaris [42].

In this study, a slightly biofilm reduction was achieved when the chitosan hydrogel treatment was applied at 0.25% (Figure 5). However, no significant reduction was achieved in the groups where aqueous solution mediated aPDT of MB was applied. When MB was incorporated into the hydrogel and associated with aPDT, we observed a reduction of up to 1.9 log_10_.

Considering that chitosan presented reduction of 1.5 log_10_ after 30 min of contact with *P. acnes* (Figure 5) and 1.9 log_10_ reduction in biofilm with aPDT (Figure 7), this cannot be considered a synergistic effect since the reductions were very similar. Although CH-MB was capable of eradicating *P. acnes* in planktonic state, this was not achieved on biofilms, so in further investigation our group will explore not only different parameters but also other photosensitizers under these conditions.

Incomplete bacterial reduction in biofilms has also been observed in many studies [6,7,36,37,40,41,43]. Our studies corroborate the literature since the main characteristic of cells organized in biofilms is the greater resistance to antimicrobials due to factors such as restricted penetration of antimicrobials, a decrease of the growth rate, expression of resistance genes and presence of persister cells [44].

In addition, work has shown that efflux pumps are highly active in bacterial biofilms and, photosensitizers such as MB, phenothiazines, are substrates of these pumps in both Gram-positive and Gram-negative bacteria [45]. These properties could justify our MB-mediated aPDT results in *P. acnes* biofilms [46].

## 4. Material and Methods

### 4.1. Materials

Methylene blue (MB), Poloxamer 407 (PO) and low-molecular-weight chitosan (CS) were purchased from Sigma Aldrich^®^ (Steinheim, North Rhine-Westphalia, Germany). Sodium hydroxide (NaOH) and glacial acetic acid were purchased from Synth^®^ (Diadema, SP, Brazil). The high-purity water was prepared with a Millipore Milli-Q Plus purification system (Molsheim, France), and its resistivity was 18.2 MΩ-cm.

### 4.2. Preparation of Hydrogels for the Structural Characterization

CS was dispersed in 0.5% (*v*/*v*) acetic acid solution at different concentrations (*w*/*v*) such as 0.25% (HG1), 0.5% (HG2) and 1.0% (HG3)and homogenized under magnetic stirrer (1000 rpm) for about 24 h. Next, the CS dispersions were kept in an ice bath to add 16% (*w*/*v*) PO under constant magnetic stirrer (1000 rpm). Then, MB was incorporated at concentrations 12.5 µg/mL (HG1-MB12.5) and 75.0 µg/mL (HG1-MB75) under stirring to ensure its complete dissolution. The pH formulations were adjusted to 6.0 with 2% (*w*/*v*) NaOH [47,48]. The formulations were stored at 4 °C and protected from light. Table 3 shows the compositions of the formulations for structural characterization.

### 4.3. Structural Characterization of the Formulations 

#### 4.3.1. Rheological Studies

Both rheological studies below were performed at room temperature (25.0 ± 0.5 °C) and skin temperature (32.0 ± 0.5 °C) in triplicate using a controlled-stress AR2000 rheometer (TA Instruments, New Castle, DE, USA) with cone-plate geometry (40 mm diameter) and a sample gap of 52 µm. The samples of the formulations were carefully applied to the lower plate to minimize the sample shearing and were allowed to equilibrate for 1 min prior to analysis [29,49].

#### 4.3.2. Determination of Flow Properties

The flow properties were determined using a controlled shear rate procedure ranging from 0.1 to 100 s^−1^ and back. Each stage lasted 120 s with an interval of 10 s between the curves. 

#### 4.3.3. Oscillatory Analyses

The oscillatory analyses were started by the conduction of a stress sweep in order to determine the viscoelastic region of the formulations. The stress sweep was carried out at a constant frequency of 1 Hz over the stress range of 0.1–10 Pa. A constant shear stress of 1.0 Pa was selected to perform the frequency sweep over a range of 0.1–10 Hz, which was within the previously determined linear viscoelastic region for all formulations. Thus, the G’ and G” moduli were recorded.

#### 4.3.4. In Vitro Bioadhesion Test

The porcine ear skin wasobtained from a slaughterhouse and prepared for the test as described by Carvalho et al. [26]. The bioadhesive force between the pig ear’s skin and the formulations was assessed by detachment test using a TA-XTplus texture analyzer (Stable Micro Systems, Surrey, UK). Before the test, the skin was attached to the lower end of a cylindrical probe (diameter 10 mm) with a rubber ring. Samples of formulations were packed into shallow cylindrical vessels and the test started lowering the analytical probe, which contained the skin at a constant speed (1 mm/s) onto the surface of the sample. The skin and the sample were kept in contact during 60 s and no force was applied during this interval. After 60 s, the skin was drawn upwards (0.5 mm/s) until the contact between the surfaces was broken. The bioadhesive force of the formulations was measured in the maximum detachment force as the resistance to the withdrawal of the probe, what reflects the bioadhesion characteristic. Five replicates were analyzed at 32.0 ± 0.5 °C.

### 4.4. Photodynamic Therapy Studies

#### 4.4.1. Bacterial Strain and Culture Medium

The model strain used in this study was *P. acnes* (ATCC 6919) obtained from the National Institute of Quality Control in Health (INCQS) from the Oswaldo Cruz Foundation (FIOCRUZ-Manguinhos, RJ, Brazil). Bacterial culture was performed using Reinforced Clostridium Agar (RCA-Himedia, Mumbai, India) culture medium supplemented with hemin. PDT tests with MB suspension were carried out with Tryptic Soy Broth medium (TSB-Acumedia, Lansing, MI, USA) for planktonic phase and supplemented with hemin when performed on biofilm. To test chitosan hydrogel with MB, Mueller-Hinton (MH) broth medium (Difco, Sparks, MD, USA) was used both in suspension and biofilm tests. 

#### 4.4.2. Development of *P. acnes* Biofilm

The biofilm development was performed according to the in vitro technique established by FONTANA et al. [6]. Prior to biofilm formation, 96-well plates were prepared with solid culture medium. To initiate biofilm growth the bacterial inoculum was adjusted to the optical density reading at 630 nm (OD_630nm_) to approximately 1 × 10^8^ cells/mL. The plate was carefully filled with 150 μL of the bacterial sample and after an initial incubation period of 48 h, the liquid medium was carefully aspirated from each well and the biofilms were replenished with fresh broth and renewed daily very slowly, to avoid disruption of the biofilm. On the seventh day of biofilm formation, the treatment was performed.

#### 4.4.3. Photosensitizer and Light Source

The phenothiazine-derivative methylene blue (MB) was the photosensitizer used in the bacterial susceptibility tests. For planktonic phase, we used concentrations of 12.5, 25.0, 37.5, 50.0 μg/mL. For biofilms, the concentrations were 75, 100 and 150 μg/mL. The light source consisted of 48 LEDs with variable intensities assembled as a compact illumination system with a homogeneous illumination area and a cooling device (IrradLED^®^—biopdi, Sao Carlos, SP, Brazil). The power density of the incident radiation was measured using a power meter (Coherent^®^, Santa Clara, CA, USA). For aPDT in planktonic phase and biofilm, *P. acnes* were incubated for 10 and 30 min in the dark respectively and then irradiated in a switched way (60 s LED on, 60 s LED off) with a system at 660 nm. Intensities of 151 mW/cm^2^ and energy densities of 90 J/cm^2^ for tests in suspension and 153 mW/cm^2^ and 150 J/cm^2^ was used in the biofilm.

#### 4.4.4. Light Absorption Spectrum of the Photosensitizer

The light absorption spectrum of the MB in solution and in the hydrogel was performed on Synergy H1M (Synergy H1 Multi-Mode Reader, Biotek, Winooski, VT, USA). A 100 μL aliquot of the sample of interest was added to a 96-well plate well and 100 μL of the blank was added at the same time in a different well, then reading was performed.

#### 4.4.5. Photodynamic Therapy

The experiments were carried out in planktonic and biofilm phases. The light was delivered uniformly to cover all 96-well of the irradiated plates. According to the power of each LED, with the dimensions of the irradiation field and the time of exposure of the light, the energy densities used was then defined. In order to avoid any kind of interference during irradiation, each plate received only one type of treatment.

#### 4.4.6. Planktonic Bacterial Susceptibility Test

Photosensitizer (PS) solution was prepared at twice the desired working concentration and 50 µL added to the correspondent wells of 96 wells plates, in triplicates. The bacterial strain, grown on agar plates in an anaerobic chamber (80% N_2_; 10% CO_2_; 10% H_2_) at 37 °C, was scraped from the agar and suspended in TSB broth at a concentration of (~1 × 10^8^ cells/mL), confirmed by spectrophotometry reading at 630 nm. Aliquots of 50 μL of the inoculum were transferred to each well of the plate, yielding a final volume of 100 µL, and the dilution of both solution and inoculum by 50%, reaching the work concentrations. Growth control was composed of 50 µL of inoculum and 50 µL of TSB or MH broth incubated for the same period as the treated groups.After incubation time and treatments, bacterial suspensions underwent serial dilutions in the same broth used in treatment groups and aliquots of 5 μL were plated on Reinforced Clostridium Agar and then incubated under anaerobic conditions for 24–48 h.

### 4.5. Statistical Analysis

Data were analyzed by one-way ANOVA with Tukey’s post-test and Variance Analysis (two-way ANOVA) with Bonferroni post-test, respectively, using Graph Pad Prism^®^ Version 5.01 software (GraphPad Software Inc., La Jolla, CA, USA). Differences were considered to be significant when *p* < 0.05 (confidence level of 95%). The maximum acceptable coefficient of variation was set at 25%.

## 5. Conclusions

Based on the results obtained in this study, it was possible to conclude that chitosan presented antimicrobial activity against *P. acnes* suspension and biofilm. However, the synergistic effect of chitosan and aPDT was observed only in the planktonic phase tests. These in vitro data suggest the potential utility of topical chitosan hydrogel as MB delivery for antimicrobial photodynamic therapy of dermatologic conditions with infectious and inflammatory components. Chitosan hydrogel is a good candidate to be doped with MB. HG1 (0.25%) was shown to be the most appropriate formulation, since higher concentrations of chitosan may increase the elasticity of the hydrogel, delaying FS release. In addition, the incorporation of MB did not significantly affect rheological and bioadhesive hydrogel’s characteristics.

## Figures and Tables

**Table 1:** Flow index (*η*) and consistency index (*k*) of HG1, HG2, HG3, HG1-MB12.5 and HG1-MB75 at room temperature (25.0 ± 0.5 °C) and at skin temperature (32.0 ± 0.5 °C).

Temperature (°C)	Formulations	Flow Index (*η)*	Consistency Index (*k*)
25 °C	HG1	0.90680 ± 0.02237	0.11022 ± 0.01066
	HG2	0.96372 ± 0.00389	0.08112 ± 0.00137
	HG3	0.92231 ± 0.00076	0.20006 ± 0.00066
32 °C	HG1	0.87836 ± 0.02885	0.10582 ± 0.01317
	HG2	0.95296 ± 0.01441	0.07338 ± 0.00459
	HG3	0.91371 ± 0.01426	0.14924 ± 0.00920
25 °C	HG1-MB12.5	0.95514 ± 0.01731	0.05200 ± 0.00390
	HG1-MB75	0.95345 ± 0.01982	0.04711 ± 0.00405
32 °C	HG1-MB12.5	0.8717 ± 0.03723	0.11857 ± 0.01904
	HG1-MB75	0.71933 ± 0.03334	0.31082 ± 0.04419

**Table 2:** Formulation strength (*S*) and viscoelastic exponent (*n*) of the formulations HG1, HG2, HG3 at room temperature (25.0 ± 0.5 °C) and skin temperature (32.0 ± 0.5 °C).

Temperature (°C)	Formulations	Formulation Strength (*S*)	Viscoelastic Exponent (*n*)
25 °C	HG1	0.00564 ± 0.00096	0.66476 ± 0.04482
	HG2	0.02120 ± 0.00395	0.34040 ± 0.05131
	HG3	0.05149 ± 0.00174	0.01859 ± 0.01020
32 °C	HG1	106.18612 ± 1.62739	0.00920 ± 0.00466
	HG2	154.58812 ± 1.65599	0.00360 ± 0.00325
	HG3	335.40866 ± 1.99548	0.00062 ± 0.00181
25 °C	HG1-MB12.5	0.98450 ± 0.47602	0.61013 ± 0.12848
	HG1-MB75	0.00152 ± 0.00089	1.78589 ± 0.14604
32 °C	HG1-MB12.5	72.48737 ± 2.79814	0.31668 ± 0.01068
	HG1-MB75	67.61907 ± 3.38183	0.32623 ± 0.01381

**Table 3:** Composition of the formulations.

Formulation	Chitosan (%) (*w*/*v*)	Poloxamer (%) (*w*/*v*)	Methylene Blue (µg/mL)
HG1	0.25	16	-
HG2	0.50	16	-
HG3	1.00	16	-
HG1-MB12.5	0.25	16	12.5
HG1-MB75	0.25	16	75
